# Habitat filtering drives the local distribution of congeneric species in a Brazilian white‐sand flooded tropical forest

**DOI:** 10.1002/ece3.7169

**Published:** 2021-01-17

**Authors:** Kelly F. O. Ribeiro, Valéria F. Martins, Thorsten Wiegand, Flavio A. M. Santos

**Affiliations:** ^1^ Programa de Pós‐Graduação em Ecologia Institute of Biology University of Campinas ‐ UNICAMP Campinas Brazil; ^2^ Department of Natural Sciences, Maths and Education Federal University of São Carlos ‐ UFSCar Araras Brazil; ^3^ Department of Plant Biology Institute of Biology University of Campinas ‐ UNICAMP Campinas Brazil; ^4^ Department of Ecological Modelling Helmholtz Centre for Environmental Research – UFZ Leipzig Germany; ^5^ German Centre for Integrative Biodiversity Research (iDiv) Halle‐Jena‐Leipzig Leipzig Germany

**Keywords:** Atlantic Forest, coexistence, community structure, congeneric species, ecological processes, habitat filtering, natural disturbance, *restinga*, spatial point processes

## Abstract

The investigation of ecological processes that maintain species coexistence is revealing in naturally disturbed environments such as the white‐sand tropical forest, which is subject to periodic flooding that might pose strong habitat filtering to tree species. Congeneric species are a good model to investigate the relative importance of ecological processes that maintain high species diversity because they tend to exploit the same limiting resources and/or have similar tolerance limits to the same environmental conditions due to their close phylogenetic relationship. We aim to find evidence for the action and relative importance of different processes hypothesized to maintain species coexistence in a white‐sand flooded forest in Brazil, taking advantage of data on the detailed spatial structure of populations of congeneric species. Individuals of three *Myrcia* species were tagged, mapped, and measured for diameter at soil height in a 1‐ha plot. We also sampled seven environmental variables in the plot. We employed several spatial point process models to investigate the possible action of habitat filtering, interspecific competition, and dispersal limitation. Habitat filtering was the most important process driving the local distribution of the three *Myrcia* species, as they showed associations, albeit of different strength, to environmental variables related to flooding. We did not detect spatial patterns, such as spatial segregation and smaller size of nearby neighbors, that would be consistent with interspecific competition among the three congeneric species and other co‐occurring species. Even though congeners were spatially independent, they responded to differences in the environment. Last, dispersal limitation only led to spatial associations of different size classes for one of the species. Given that white‐sand flooded forests are highly threatened in Brazil, the preservation of their different habitats is of utmost importance to the maintenance of high species richness, as flooding drives the distribution of species in the community.

## Graphical abstract

We aim to find evidence for the action and relative importance of different ecological processes hypothesized to maintain species coexistence in a tropical forest subject to seasonal flooding, using the spatial structure of populations of three congeneric species. We collected environmental data and data on trees of three *Myrcia* species within a 1‐ha plot of a Brazilian white‐sand flooded tropical forest. We find that habitat filtering to areas subject to flooding is the most important ecological process driving the local distribution of the species studied in a white‐sand forest.

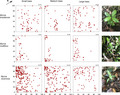

## 1 INTRODUCTION

Understanding the ecological processes that maintain high species diversity in natural environments, such as tropical forests, is fundamental in community ecology (Brown, 2014; Leigh et al., 2004). The main processes maintaining species coexistence in tropical forests are deterministic or stochastic (Chase & Myers, 2011). The deterministic processes of interspecific competition and habitat filtering are based on the species’ niche (Wright, 2002). Species that exploit the same limiting resources or that have similar tolerance limits to the same environmental conditions compete intensely (Chesson, 2000), especially in the case of sessile organisms such as trees (Silvertown, 2004). In a two‐species model with asymmetric competition, the stronger species exclude the weaker species (Schluter, 2000) or constrain the spatial distribution of the weaker species to less favorable environments (Baraloto et al., 2007). Thus, if these deterministic processes are important forces structuring a community, species coexistence is possible when there is niche partitioning among the species or high environmental heterogeneity that enables species with similar niches to be restricted to somewhat different environments (Silvertown, 2004). On the other hand, according to the neutral theory (Hubbell, 2001), species diversity results from a balance between stochastic emergence and disappearance of species at the regional scale (Hubbell, 2005). Locally, seed arrival in vacant recruitment sites is unpredictable, considering the spatio‐temporal variation in availability of space. However, dispersal and recruitment limitation, common in tropical forests (Hubbell et al., 1999), can prevent the most abundant species from occupying all available recruitment sites and thus dominating the community over time (competition exclusion), that is, “winning‐by‐forfeit” (Hubbell, 2001; Hurtt & Pacala, 1995).

The ecological processes that act on each individual plant throughout its life determine the arrangement of plants in space, with each process generating a characteristic spatial structure at a given spatial scale (Hubbell et al., 2001). Habitat filtering, for example, is expected to result both in spatial segregation of species with different environmental requirements at spatial scales larger than the size of patches within which the environment is approximately homogeneous (Getzin et al., 2006; Itoh et al., 2003; Table 1) and in spatial association of species with similar environmental requirements at spatial scales smaller than these patch scale (Burns & Strauss, 2011; Table 1). When these species additionally compete within patches, reduced resource availability should result in a decrease in growth rate (Kenkel, 1988), thereby causing nearby neighbors to be smaller than distant trees (Getzin et al., 2008; Table 1). Also, in extreme cases, the stronger competitor can eventually cause mortality of the weaker competitor (Kenkel, 1988), resulting in spatial segregation of competing species at the small neighborhood scale (say < 5 m; Velázquez et al., 2015; Table 1).

**TABLE 1:** Expectations for different ecological processes driving local species distribution

Questions/Ecological process driving species distribution	Expectation for different ecological processes				Analysis	Results		
	Species have different environmental requirements and do not compete	Species have the same environmental requirements and do not compete	Species have the same environmental requirements and compete	Species patterns according to neutral theory		*Myrcia brasiliensis*	*Myrcia multiflora*	*Myrcia racemosa*
Which environmental variables are associated with the spatial distribution of the different size classes of each species?	Congeners distribution is related to different environmental variables, especially in large trees	Congeners distribution is related to the same environmental variables, especially in large trees	Congeners distribution is related to the same environmental variables, especially in large trees	No relationship between species distribution and environmental variables	Log‐linear regression model	Small trees: relationship to higher canopy openness in 2017 and 2008	Small trees: relationship to lower terrain slope and elevation, and higher soil moisture and canopy openness in 2008	Small trees: relationship to lower elevation and terrain slope
						Large trees: relationship to lower terrain slope and elevation	Medium trees: relationship to lower terrain slope and elevation, and higher canopy openness in 2008	Medium trees: relationship to lower elevation, and higher canopy openness in 2008
							Large trees: relationship to lower terrain slope, elevation, and litterfall height, and higher soil moisture	Large trees: relationship to lower canopy openness in 2008
What is the spatial relationship between congeners in the same size class?	Spatial segregation of congeners at the patch scale, especially for large trees	Spatial association of congeners at the scale of suitable patches, especially for large trees	Spatial association of congeners at the patch scale, especially for large trees, and tendency to segregation at the neighborhood scale	Spatial independence of congeners in all size classes	*g_12_*(*r*) with pattern reconstruction of species 1 with spatially constant intensity *λ* for the independence null model, and with the parametric *λ_h_*(*x*,*y*) for testing environmental filtering	Spatial independence of congeners in all size classes	Spatial independence of congeners in all size classes	Spatial independence of congeners in all size classes
Is the size of an individual influenced by the proximity to congeners in the same size class?	No influence of nearby congeners on tree size	No influence of nearby congeners on tree size	Negative influence of nearby congeners on tree size	No influence of nearby congeners on tree size	*κ_m_*_1_.(*r*) with the null models of regular random marking for a general analysis and local random marking for specific test of environmental filtering	No influence of nearby congeners on tree size	No influence of nearby congeners on small and large trees. Medium trees was influenced by less favorable environmental conditions	No influence of nearby congeners on small and large trees. Medium trees was influenced by less favorable environmental conditions
What is the spatial relationship between small and large conspecific trees?	Spatial association of small and large trees due to habitat filtering and/or dispersal limitation	Spatial association of small and large trees due to habitat filtering and/or dispersal limitation	Spatial association of small and large trees due to habitat filtering and/or dispersal limitation	Spatial association of small and large trees due to dispersal limitation	A suit of competing point process models to distinguish among independence and association due to dispersal limitation and/or shared habitat associations	Spatial independence of conspecifics in all size classes	Spatial association small and large trees due to dispersal limitation; spatial association of medium and large trees due to the combination of dispersal limitation and habitat filtering	Spatial independence of conspecifics in all size classes
Are the spatial patterns of the three congeners explained by interactions with other co‐occurring species?	Spatial segregation between each *Myrcia* species and heterospecifics at the patch scale for large trees	Spatial association between each *Myrcia* species and heterospecifics at the scale of suitable patches for large trees	Spatial association between each *Myrcia* species and heterospecifics at the patch scale for large trees, and tendency to segregation at the neighborhood scale	Spatial independence between each *Myrcia* species and heterospecifics in all size classes	*g_12_*(*r*) with pattern reconstruction of species 1 with spatially constant intensity *λ* for the independence null model, and with the parametric *λ_h_*(*x*,*y*) for testing environmental filtering	Spatial independence between each *Myrcia* species and heterospecifics in all size classes	Spatial independence between each *Myrcia* species and heterospecifics in all size classes	Spatial independence between each *Myrcia* species and heterospecifics in all size classes

Note

The analyses employed in this study and the results for three size classes of three *Myrcia* species sampled in a 1‐ha plot of white‐sand flooded forest, southeastern Brazil, are also shown. The neighborhood scale (up to some 10 m) represents direct species interactions, the patch scale represents approximately homogeneous (suitable or less suitable) environments, and the community scale (100 m—tens of km) comprises several patches.

In contrast, if stochastic mechanisms govern community structuring as assumed in the neutral theory, we should observe an approximate independent spatial relationship between species pairs (Volkov et al., 2007; Table 1). Additionally, species distributions should not be related to environmental characteristics, as all species are expected to respond to the environment in a similar way (Hubbell, 2005). Last, dispersal limitation is expected to result in aggregation of seeds and small trees, and in spatial associations of seeds and smaller trees with large trees (Murphy et al., 2017; Table 1).

Even though habitat filtering and dispersal limitation both result in aggregation and spatial association of conspecifics, the former reflects environmental tolerance of species (Kraft et al., 2015) that can lead to species distribution related to local environmental variables, whereas the latter can cause high density of small trees centered on conspecific large trees (Wang et al., 2015). Additionally, the spatial pattern of conspecifics of different sizes can indicate which ecological process has acted more strongly on the population (Comita et al., 2007; Shen et al., 2009). Species–habitat association might change across demographic stages of plant development, possibly resulting in spatial dissociation between size classes. Moreover, species–habitat association is expected to be stronger for large than for small trees (Comita et al., 2007; but see Baldeck et al., 2013). On the other hand, aggregation of conspecifics of different sizes and spatial association between size classes are expected under strong dispersal limitation (Wiegand et al., 2007). However, spatial patterns driven by dispersal limitation may be masked by high mortality of seeds and small trees that occur at high local densities in the neighborhood of parent trees due to intraspecific competition and the attack of species‐specific natural enemies (Comita et al., 2014; Getzin et al., 2014), which is known as conspecific negative‐density dependence (CNDD; LaManna et al., 2017).

The investigation of ecological processes that maintain species coexistence is revealing in naturally disturbed environments such as the Brazilian white‐sand tropical forest, which is subject to periodic flooding (Eisenlohr et al., 2013). Flooding reduces the amount of oxygen available for roots, and consequently limits plant growth (Oliveira & Joly, 2010) and increases plant mortality (Johnson et al., 2017). The soil moisture gradient thus acts as a selective pressure on plants that can result in niche differentiation among co‐occurring species (Werner & Platt, 1976) and different species–habitat associations in the white‐sand tropical forest, according to each species’ flooding tolerance and local environmental heterogeneity (Baraloto et al., 2007). Additionally, flooding can act as a strong driver of species composition through habitat filtering (Baraloto et al., 2007; Oliveira et al., 2014; Eisenlohr et al., 2013). Therefore, changes in environments subject to flooding, such as those caused by climate change or land use changes, are likely to lead to drastic changes in community structure. These changes include spatial rearrangement or local extinction of species according to their tolerance ranges and the new environmental characteristics (Kraft et al., 2015). Nevertheless, few studies have evaluated the spatial structure of trees in environments with seasonal flooding such as the highly threatened Brazilian white‐sand tropical forest (e.g., Baraloto et al., 2007; Oliveira et al., 2014).

Congeneric species are a good model to investigate the relative importance of ecological processes that maintain high species diversity because they tend to exploit the same limiting resources and/or have similar tolerance limits to the same environmental conditions due to their close phylogenetic relationship (Losos, 2008). Therefore, strong interspecific competition within the same tolerable environment should be easily detected, resulting in spatial association between species at the scale of environmental patches (< 30 m) due to habitat filtering and dissociation at the scale of local neighborhood (< 5 m) due to interspecific competition (Velázquez et al., 2015). However, congeneric species may show niche differentiation due to selective pressures that act on each one of them at the evolutionary scale, including competition within the same tolerable environment (Wiegand et al., 2007). This “ghost of competition past” results, at present day, in each species specialized in a different environment (Stubbs & Wilson, 2004; Yamada et al., 2005) or in different use of resources within the same tolerable environment (Schluter, 2000). Alternatively, species’ niche may not be important, and so the spatial structure of populations should reflect dispersal limitation and stochasticity (May et al., 2014).

The present study aims to find evidence for the action and relative importance of different ecological processes hypothesized to maintain species coexistence in a tropical forest subject to seasonal flooding, taking advantage of data on the detailed spatial structure of populations of three congeneric species. Specifically, we ask: (a) Which environmental variables are associated with the spatial distribution of the different size classes of each species? (b) What is the spatial relationship between congeners in the same size class? (c) Is the size of an individual influenced by the proximity to congeners in the same size class? (d) What is the spatial relationship between small and large conspecific trees? (e) Are the spatial patterns of the three congeners explained by interactions with other co‐occurring species? The combination of different spatial patterns will indicate which ecological processes are more important to the maintenance of species coexistence in the white‐sand flooded tropical forest (see Table 1).

## 2 MATERIAL AND METHODS

### 2.1 Study site

The Brazilian Atlantic Forest covers today only about 12% of its original extension and is distributed in fragments (Ribeiro et al., 2009). Because it also presents high species richness and endemism, the forest along the coast was classified as a hotspot for biodiversity conservation (Myers et al., 2000). The vegetation closest to the beach, covering sandy soils, is called *restinga*. It can vary from sparse herbaceous to forest communities (i.e., white‐sand tropical forest; Oliveira et al., 2014). The low altitude (<10 m; Joly et al., 2012) and fairly shallow water table result in periodic flooding, especially during the rainy season. Microtopographic variation forms dry sandy cords between flood channels, which, when dry, hold high quantities of organic matter (Diniz, 2009). While some plant species are flood‐tolerant, others are restricted to the sandy cords (Oliveira et al., 2014). Due to their proximity to the beach, *restinga* communities are highly threatened in Brazil by the real estate market (Alho et al., 2002).

In the present study, we collected data in a 1‐ha, permanent plot (23°21′22″S and 44°51′03″W) installed at a protected white‐sand flooded forest in the northern coast of the state of São Paulo, southeastern Brazil. There is a mountain range along the coast and the forest studied was recently formed from the downward movement of some species from the Atlantic Forest of the interior of the state of São Paulo (Eisenlohr et al., 2013; Sanchez et al., 2013). The white‐sand forest is composed of dense vegetation with a large proportion of trees in the intermediate stratum (5–10 m height), but light levels are high in the understory due to canopy irregularities. There are also a shrub and an herbaceous stratum, epiphytes and lianas (Joly et al., 2012). The regional climate is tropical humid, with mean annual precipitation of 2,634 mm and mean annual temperature around 22°C (Morellato et al., 2000). The study plot was divided into 100 subplots of 10 × 10 m and presents 84 tree and palm species, being Myrtaceae and Fabaceae the richest families, and Myrtaceae, Arecaceae, and Euphorbiaceae the most abundant ones (Joly et al., 2012).

### 2.2 Species studied

The three species studied belong to the genus *Myrcia*, family Myrtaceae, subfamily Myrtoideae. Myrtaceae presents the highest number of species in tropical rainforests (Oliveira‐Filho & Fontes, 2000) and is considered a characteristic family in the Brazilian Atlantic coastal forest (Lucas & Bünger, 2015). In the study plot, there are 21 Myrtaceae species, seven belonging to *Myrcia* (Joly et al., 2012). The three species studied have 50 or more individuals in the plot (individuals with stem diameter at breast height ≥ 4.8 cm; data from the Functional Gradient Project Biota/FAPESP 03/12595‐7—available upon request).

The three species are not restricted to the white‐sand forest and are widely distributed in the Atlantic Forest (Appendix S1). Their fruits have a fleshy pulp with 1–4 seeds and dark‐reddish coloration, characteristics attractive to birds (Castro, 1998; Pedroni, 2001). Fruits of *Myrcia brasiliensis* show a larger diameter (9–14 mm; Caliari, 2013) than fruits of *Myrcia multiflora* and *Myrcia racemosa* (3.5–6.5 mm; Morais & Lombardi, 2006; Souza et al., 2007; Rosa & Romero, 2012). In the study plot, the species occupy different canopy strata. *Myrcia brasiliensis* occupies the first stratum and also occurs as an emergent tree; *M. multiflora* occupies the first and second strata, and *M. racemosa* occupies the first stratum (Pedroni, 2001). The three species increase water absorption in the soil at 50 cm depth and reduce the use of topsoil water (10 cm depth) from the flooded to the dry period. In the dry period, the species show a similar use of water sources, but *M. racemosa* absorbs a higher proportion of water at 30 cm depth (Antunes, 2018). The similar water use suggests that the three *Myrcia* species are restricted to similar environments in the periodically flooded white‐sand forest studied here, but we lack additional data on between‐species variation of functional traits and plant physiological processes to hypothesize whether the species occupy similar niches.

### 2.3 Data collection

Between 2016 and 2017, we marked with individually numbered tags all freestanding stems with diameter at soil height (DSH) ≥ 1.5 mm (minimum size at which taxonomic identification was possible) of the three species studied. We also measured the DSH and mapped the *x*, *y* coordinates of all individuals in the study plot. Diameter at soil height was measured above root insertion, at the point where the stem shows an approximated circular shape.

We sampled environmental variables that are likely related to different species requirements in a white‐sand flooded tropical forest. Canopy openness, elevation, terrain slope, and soil moisture were obtained at a 10 × 10 m scale, while flooding depth and litterfall depth were measured at a 5 × 5 m scale. We measured canopy openness, elevation, and terrain slope following Rosa et al. (2019). Canopy openness was calculated from hemispherical photographs and represents light availability in the understory. Because recent light environment should influence smaller plants and past light environment likely influenced large trees as they were growing (Poorter, 2007), we measured canopy openness at two different times (years 2008 and 2017). Elevation and terrain slope are associated with flooding depth in the study area, with lower flooding depth in higher and steeper patches (Diniz, 2009). Soil moisture was determined as the volumetric soil water content measured at the center of each subplot at 12 cm depth with a HS2 Hydrosense II sensor (Campbell Scientific; Uriarte et al., 2018) and represents soil water availability. We measured flooding depth and litterfall depth at four diagonal points 3.5 m from each corner of the subplots using a graduated stake (Souza & Martins, 2005; Wang & Augspurger, 2006). Flooding depth was measured during the rainy season (January 2017), as flooding can limit plant growth (Oliveira & Joly, 2010) and increase plant mortality (Johnson et al., 2017). Although litterfall is not related to the environmental requirements of plants, it can cause seedling mortality and significantly influence forest regeneration (Gillman, 2016).

In order to determine whether the spatial patterns of the three *Myrcia* species were explained by interactions with other co‐occurring species, we selected the species with 30 or more individuals in the study plot (abundant species) from the Functional Gradient Project database to be included in our analysis. We only included abundant species because smaller samples result in too large stochastic effects in the characterization of the spatial structure of populations (Wiegand et al., 2016). The database presents botanical identification, diameter at breast height (DBH) and spatial location (*x*, *y* coordinates) of each individual tree or palm with DBH ≥ 4.8 cm (Joly et al., 2012). Our dataset comprised 14 abundant species including the three *Myrcia* species studied (Appendix S2).

### 2.4 Data analysis

#### 2.4.1 Size classes of the three *Myrcia* species

To investigate ecological processes across demographic stages of tree development, we divided the individuals of each of the three *Myrcia* species into three size classes based on DSH and taking into account the minimum number of individuals needed in each class for spatial point pattern analysis (*n* ≥ 30, Martins et al., 2018). The smaller size class limit differs between *M. brasiliensis* and the other two species. Small trees were defined as individuals with DSH ≤ 0.2 cm for *M. brasiliensis*, and DSH ≤ 0.5 cm for *M. multiflora* and *M. racemosa*. Medium‐sized trees had 0.2 < DSH ≤ 10.0 cm for *M. brasiliensis*, and 0.5 < DSH ≤ 10.0 cm for *M. multiflora* and *M. racemosa*. Large trees were those with DSH > 10.0 cm for the three species. We conducted all the following analyses separately for the three size classes of each of the three species studied.

#### 2.4.2 Analysis 1: Relationship between the distribution of the three *Myrcia* species and environmental variables

To test for species–habitat association across demographic stages of plant development, we first used the variance inflation factor (VIF) to check whether our seven environmental variables were multicollinear (VIF > 10, Zuur et al., 2007). None of them showed multicollinearity; we therefore included all environmental variables in our models. We modeled the intensity function *λ_h_*(*x*, *y*) at the location (*x*, *y*) as a function of the sum of the variables on a log scale. The corresponding log‐linear regression model yields:

$$\text{log}(\lambda_{h}(x,y))=\beta_{0}+\beta_{1}v_{1}(x,y)+\ldots +\beta_{7}v_{7}(x,y),$$

where *β*_0_ is the intercept and *β*_i_ is the coefficient of the *i*th environmental variable *v*_i_(*x*, *y*) to be estimated (Waagepetersen, 2007), with *v*_1_ being flooding depth, *v*_2_ soil moisture, *v*_3_ terrain slope, *v*_4_ elevation, *v*_5_ litterfall depth, *v*_6_ canopy openness in 2017, and *v*_7_ canopy openness in 2008.

We used the *Z* value to evaluate the significance and direction of the effect of the different environmental variables on each species’ pattern. For a significance level of *α* = 0.05, we have a significant and positive association with a given variable if *Z* > 1.96 (and negative if *Z* < −1.96) and the larger the absolute value of *Z*, the stronger the association. We considered habitat effects to be strong when *Z* ≥ |4|. We fitted *λ_h_*(*x*, *y*) using maximum likelihood estimation to determine the values of the coefficients *β*_i_ (Shen et al., 2009; Wang et al., 2011) in the package “spatstat” (Baddeley et al., 2015) of the software R (R Core Team, 2018).

#### 2.4.3 Analysis 2: Spatial relationship between *Myrcia* species and between each *Myrcia* species and heterospecifics

Here, we investigated the spatial relationship between the individuals of two congeners of the same size class. Additionally, we apply the same methods to assess spatial relationships between large trees of each of the three *Myrcia* species and co‐occurring abundant species. To this end, we used the bivariate pair correlation function *g*_12_(*r*) as the summary function (Stoyan & Stoyan, 1994) and the null model of spatial independence, in which we fixed the original location of points of pattern 2 and randomized the point pattern of focal species 1. We used two different null models to generate null model patterns of the focal species that conserve its univariate pattern. They are based on the technique of pattern reconstruction (Wiegand et al., 2013) and use a spatially constant intensity function *λ* (i.e., homogeneous pattern reconstruction) and the intensity function *λ_h_*(*x*, *y*) (i.e., inhomogeneous pattern reconstruction) describing the environmental dependency of the focal species (Wang et al., 2015; Wiegand et al., 2013).

Positive departures of the observed *g*_12_(*r*) from the homogeneous null model indicate species association (caused by positive interspecific interactions or the location of the two species within the same environmental patches), negative departures indicate species dissociation (caused by negative interspecific interactions such as competition or the location of the two species in different environmental patches), and values within the simulation envelope indicate no significant departure from independence (Wiegand & Moloney, 2014). Departures from the inhomogeneous null model suggest operation of species interactions beyond habitat association quantified by *λ_h_*(*x*, *y*) or missing environmental variables (Wiegand & Moloney, 2014).

#### 2.4.4 Analysis 3: Spatial distribution of size between *Myrcia* species and between each *Myrcia* species and heterospecifics

Here, we are interested in assessing if the size of an individual of a focal species 1 is influenced by the proximity to congeners of a species 2 in the same size class. Additionally, we search for interactions between large trees of each of the three *Myrcia* species (species 1) and the co‐occurring abundant species in the study plot (species 2). For this, we used the bivariate *r*‐mark correlation function with our size measure DSH as the quantitative mark. The bivariate *r*‐mark correlation function *κ_m_*.(*r*) measures in our case the mean size of focal trees of species 1 that have a species 2 neighbor located at distance *r*. This function is normalized by the mean size of all individuals of the focal species 1. Because *κ_m_*_1_.(*r*) is evaluated at different interpoint distances *r*, we can observe how spatial effects on the quantitative mark decline with increasing distance to individuals of species 2 (Wiegand & Moloney, 2014).

In order to generate the null model values representing the absence of interactions for comparison of *κ_m_*_1_.(*r*), we randomly shuffled the size (i.e., DSH) among individuals of the focal species 1; additionally, the original location of all individuals was kept fixed. This “random marking” null model assumes that the size of the focal individuals is independent of the size of nearby individuals of species 2. Positive departures from the observed *κ_m_*.(*r*) indicate positive interspecific interactions or both species are located at favorable patches, whereas negative departures indicate negative interspecific interactions such as competition, or location of the two species at unfavorable patches (Wiegand & Moloney, 2014).

In order to separate the effects of environmental heterogeneity and the effects interspecific interactions, we used the local random marking null model. In this variation of the null model, shuffling of the sizes of the focal species 1 is restricted to points separated only up to a given distance *R*, which represents an approximated size of environmental patches. By doing this, we can check whether departures from the independent marking null model are caused by environmental heterogeneity. Conversely, departures from the local random marking indicate interspecific interactions within patches (Wiegand & Moloney, 2014). We stipulated *R* = 25 m (one‐quarter of the 1‐ha plot) for the local random marking null model after testing for different values of *R*. The selected value corresponds to an approximate patch size where most distance effects disappear. Additionally, *R* = 25 m is large enough to encompass neighborhood interactions within patches. In all analyses, we considered the influence of species 1 on species 2 and vice versa.

#### 2.4.5 Significance of spatial patterns against the null models for analysis 2 and 3

In order to determine whether the observed summary functions indicating spatial associations between species (*g*_12_(*r*)) and the spatial distribution of size between species (*κ_m_*_1_.(*r*)) differed from the expected values under the appropriate null models, we first performed 199 Monte Carlo simulations of the point processes underlying the null models. Then, we created global simulation envelopes for the 1–50 m distance interval, considering the critical *α* value as calculated in the Z test statistics. The global envelopes have the convenient property that the null model can be rejected with the prescribed significance level *α* if the observed summary function falls at least at one distance bin outside the envelopes (Wiegand et al., 2016).

#### 2.4.6 Analysis 4: Spatial relationship between *Myrcia* conspecifics of different sizes

In order to assess ecological processes driving spatial relationships between *Myrcia* conspecifics of different size classes, we used a suite of alternative point process models that represent competing hypotheses, namely, independence, dispersal limitation, habitat filtering, and a combination of dispersal limitation and habitat filtering. In all cases, we fixed the pattern of large trees, and randomized the pattern of small and medium trees according to the point process model used. To conserve the univariate pattern of small and medium trees during the simulations, we again used the technique of pattern reconstruction (Wiegand et al., 2013) with different intensity functions tailored specifically for each point process model.

For the independence hypothesis (lack of small‐scale species interactions), we used a spatially constant intensity function *λ* (i.e., homogeneous pattern reconstruction as in analysis 2). To represent dispersal limitation, we used the intensity function *λ_d_*(*x*, *y*) given by the superposition of Gaussian kernels with parameter *σ* around large trees. This creates patterns where the distribution of small and medium trees follows a normal distribution around the large conspecific trees (Wiegand & Moloney, 2014). The value of the parameter *σ* was fitted. For the habitat association hypothesis, we used the parametric intensity function *λ_h_*(*x*, *y*) of small trees as in analysis 1. Finally, to represent the combined dispersal limitation and habitat filtering hypothesis, we used the geometric mean of the two intensity functions (i.e., [*λ_d_*(*x*, *y*) *λ_h_*(*x*, *y*)]^0.5^).

To determine the most parsimonious point process model, given the data, we used model selection based on the Akaike information criterion (AIC) and “synthetic” likelihood functions (Wood, 2010). With this method, we reduced the raw point pattern data to three‐point pattern summary functions that quantify the spatial structure of the observed bivariate point patterns, namely *g*_12_(*r*), the bivariate *L*‐function *L*_12_(*r*), and the bivariate nearest neighbor distribution function *D*_12_(*r*). We performed 999 simulations of the point process model to obtain the mean and the covariance matrix of these functions for each radius *r* (in steps of 3 m), given the vector **θ** of model parameters. This allows for the construction of the synthetic likelihood to assess model fit. The resulting log‐likelihood can then be used to calculate the AIC that balances model fit and model complexity to identify the most parsimonious model (Akaike, 1974; Wiegand & Moloney, 2014). We used here 999 simulations for better estimation of the covariance matrix needed for construction of the likelihood function.

We performed all point pattern analyses with the software *Programita* (Wiegand & Moloney, 2014), which can be accessed at www.programita.org. Estimators of the summary functions and the edge correction used in *Programita* are detailed in Wiegand et al. (2016). We used a spatial resolution of 1 m, which is much smaller than the study plot, fine enough to answer our questions, and larger than the mapping error of the data (Wiegand & Moloney, 2004, 2014). We selected a ring width *d* = 3 m in all analyses.

## 3 RESULTS

### 3.1 Number of *Myrcia* individuals and distribution of size classes in the plot

**FIGURE 1:**
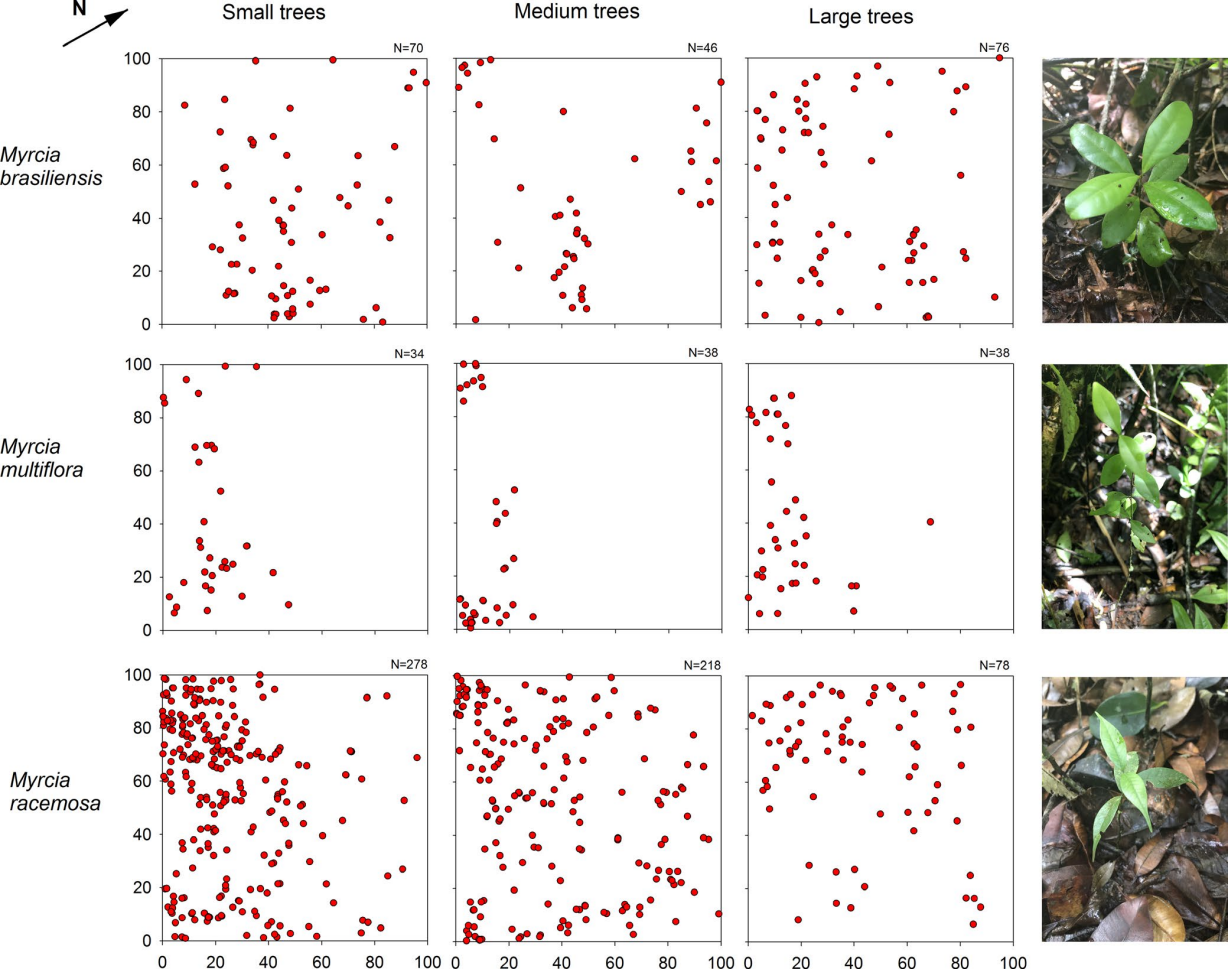
Distribution of individuals of different size classes of three *Myrcia* species in a 1‐ha plot of white‐sand flooded forest, southeastern Brazil. For size thresholds, please see the text. The photographs show an individual of each of the three *Myrcia* species; photographs taken by Kelly Ribeiro

**FIGURE 2:**
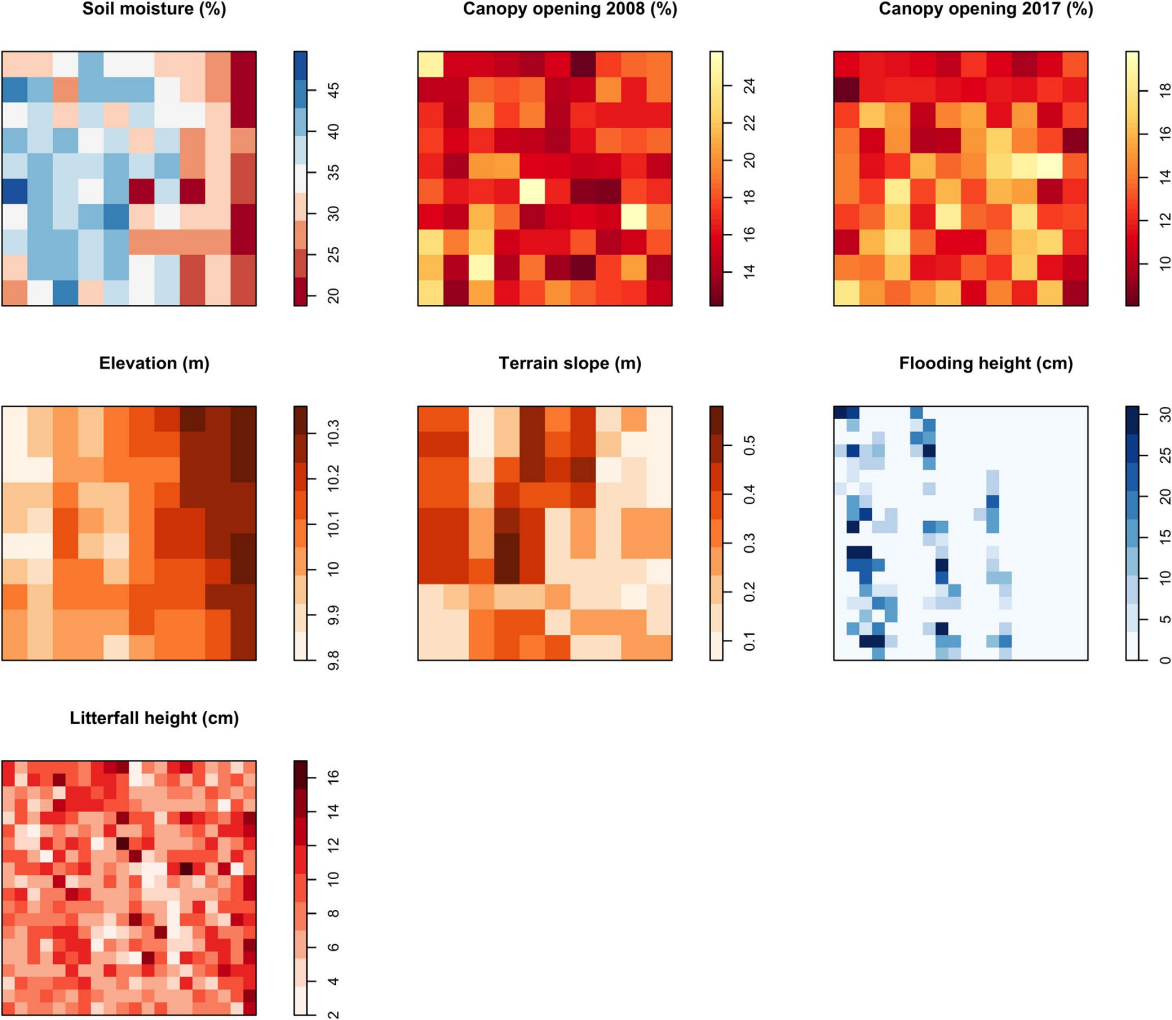
Maps of environmental variables measured in a 1‐ha plot of white‐sand flooded forest, southeastern Brazil

Our three focal species showed different abundances and distribution patterns within the plot. *Myrcia racemosa* showed the highest number of individuals in all size classes (*n* = 574), followed by *M. brasiliensis* (*n* = 192) and *M. multiflora* (*n* = 110, Figure 1). Individuals of all size classes of *M. brasiliensis* were well distributed across the study plot. Small and medium trees of *M. racemosa* were distributed as a decreasing gradient from flooded to drier areas (from northwest to southeast of the plot), and large trees were distributed as a decreasing gradient from north to south of the plot. All size classes of *M. multiflora* occurred only in the flooded area (west part of the plot; Figures 1 and 2).

### 3.2 Relationship between the distribution of the three *Myrcia* species and environmental variables

Results of this analysis are summarized in Table 2. On overall, the three species occur in similar environments in the plot, that is, low flat areas and better‐lit patches. For *M. brasiliensis*, small trees were associated to areas with higher canopy openness in 2017 and 2008, while large trees showed associations with lower terrain slope and elevation. However, these associations were weak. All size classes of *M. multiflora* were related to lower terrain slope and elevation. Additionally, small and large *M. multiflora* trees were associated with higher soil moisture, small and medium trees were associated with higher canopy openness in 2008, and large trees were related to lower litterfall depth. Elevation was the environmental variable that more strongly influenced the distribution of individuals, especially of medium and large trees. The distribution of small and mainly medium trees of *M. racemosa* was also strongly influenced by lower elevation. Additionally, small trees were strongly related to lower terrain slope, and medium trees, weakly and positively related to canopy openness in 2008, which kept influencing the distribution of large trees, but now in a negative fashion.

**TABLE 2:** Results of species–habitat association analysis using log‐linear regression models for different size classes three *Myrcia* species sampled in a 1‐ha plot of white‐sand flooded forest, southeastern Brazil

*M. brasiliensis*	Estimate	*SE*	*Z* value	*M. multiflora*	Estimate	*SE*	*Z* value	*M. racemosa*	Estimate	*SE*	*Z* value
Small trees				Small trees				Small trees			
(Intercept)	−5.51	13.70	−0.40	(Intercept)	70.70	21.29	3.32***	(Intercept)	77.23	6.75	11.44***
Flooding depth	−0.01	0.02	−0.42	Flooding depth	0.00	0.02	0.04	Flooding depth	0.00	0.01	−0.14
Soil moisture	0.05	0.03	1.92	**Soil moisture**	**0.09**	**0.04**	**2.53** *	Soil moisture	−0.01	0.01	−0.45
Terrain slope	−0.16	1.19	−0.13	**Terrain slope**	**−5.01**	**1.89**	**−2.65** **	**Terrain slope**	**−2.04**	**0.61**	**−3.34** ***
Elevation	−0.43	1.31	−0.33	**Elevation**	**−8.03**	**2.09**	**−3.84** ***	**Elevation**	**−7.99**	**0.65**	**−12.24** ***
Litterfall depth	0.04	0.05	0.85	Litterfall depth	0.00	0.07	0.02	Litterfall depth	0.04	0.02	1.84
**Canopy openness 2017**	**0.10**	**0.05**	**2.02** *	Canopy openness 2017	0.02	0.08	0.23	Canopy openness 2017	−0.05	0.03	−1.93
**Canopy openness 2008**	**0.08**	**0.04**	**2.29** *	**Canopy openness 2008**	**0.11**	**0.05**	**2.09** *	Canopy openness 2008	0.03	0.02	1.38
Medium trees				Medium trees				Medium trees			
(Intercept)	−0.89	16.50	−0.05	(Intercept)	171.92	33.27	5.17***	(Intercept)	50.38	7.28	6.92***
Flooding depth	0.02	0.02	1.35	Flooding depth	0.03	0.02	1.50	Flooding depth	0.00	0.01	−0.23
Soil moisture	0.01	0.03	0.26	Soil moisture	0.02	0.04	0.50	Soil moisture	−0.02	0.01	−1.23
Terrain slope	1.96	1.41	1.39	**Terrain slope**	**−11.96**	**2.72**	**−4.40** ***	Terrain slope	−1.08	0.67	−1.60
Elevation	−0.64	1.57	−0.41	**Elevation**	**−18.18**	**3.39**	**−5.36** ***	**Elevation**	**−5.38**	**0.70**	**−7.71** ***
Litterfall depth	−0.02	0.06	−0.40	Litterfall depth	−0.01	0.07	−0.20	Litterfall depth	0.00	0.03	−0.18
Canopy openness 2017	−0.03	0.06	−0.49	Canopy openness 2017	0.12	0.07	1.60	Canopy openness 2017	−0.02	0.03	−0.66
Canopy openness 2008	0.09	0.05	1.95	**Canopy openness 2008**	**0.26**	**0.06**	**4.54** ***	**Canopy openness 2008**	**0.06**	**0.02**	**3.03** ***
Large trees				Large trees				Large trees			
(Intercept)	22.95	12.62	1.82	(Intercept)	83.07	20.09	4.13***	(Intercept)	7.68	13.10	0.59
Flooding depth	0.00	0.02	−0.20	Flooding depth	0.01	0.02	0.69	Flooding depth	0.02	0.01	1.53
Soil moisture	0.04	0.02	1.51	**Soil moisture**	**0.08**	**0.04**	**2.24** *	Soil moisture	0.03	0.03	1.25
**Terrain slope**	**−2.57**	**1.15**	**−2.23** *	**Terrain slope**	**−4.25**	**1.78**	**−2.38** *	Terrain slope	0.23	1.09	0.21
**Elevation**	**−2.92**	**1.21**	**−2.42** *	**Elevation**	**−8.86**	**1.97**	**−4.51** ***	Elevation	−1.13	1.24	−0.91
Litterfall depth	0.02	0.04	0.52	**Litterfall depth**	**−0.17**	**0.07**	**−2.34** *	Litterfall depth	0.04	0.04	0.91
Canopy openness 2017	0.03	0.05	0.64	Canopy openness 2017	−0.08	0.07	−1.11	Canopy openness 2017	−0.08	0.05	−1.58
Canopy openness 2008	0.02	0.04	0.65	Canopy openness 2008	0.03	0.05	0.64	**Canopy openness 2008**	**−0.11**	**0.05**	**−2.28** *

Note

Bold indicates significant species–habitat association.

* *p* < 0.05.

** *p* < 0.01.

*** *p* < 0.001.

### 3.3 Spatial relationship between *Myrcia* species

**FIGURE 3:**
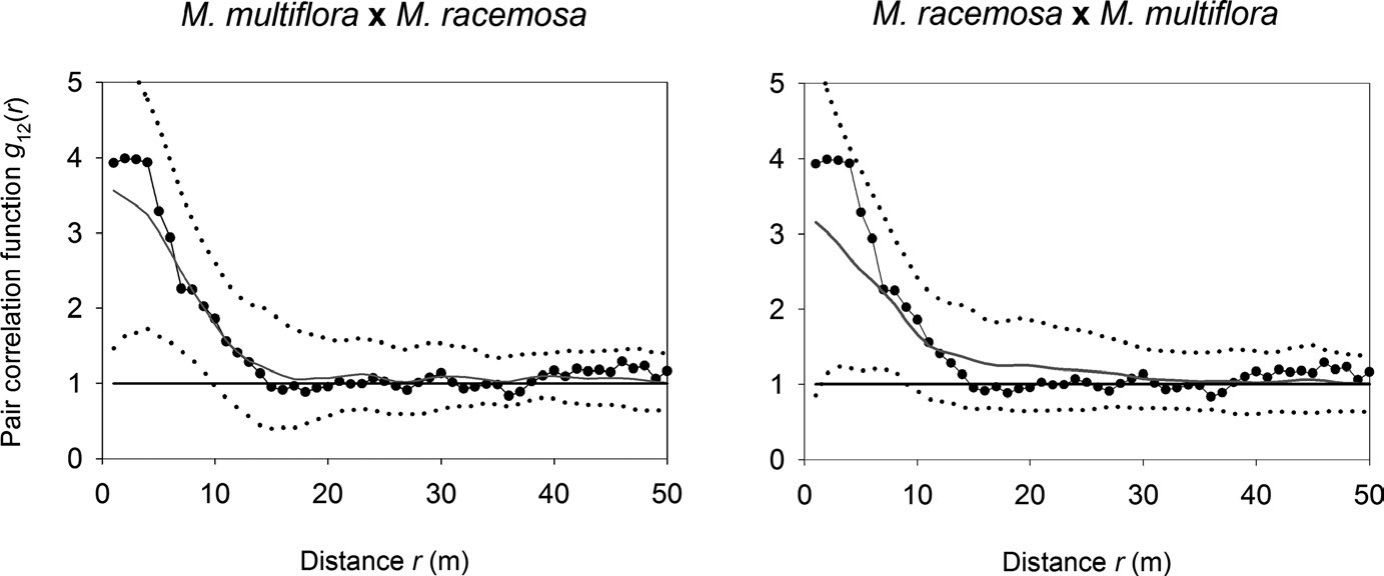
Spatial relationship, as measured by the bivariate pair correlation function *g*_12_(*r*), between medium trees of *Myrcia multiflora* and *Myrcia racemosa* (pairwise) sampled in a 1‐ha plot of white‐sand flooded forest, southeastern Brazil. The spatial relationship was compared to a null model representing large‐scale habitat association. The observed *g*_12_(*r*) is represented by closed circles, the mean *g*_12_(*r*) of 199 simulations by gray solid lines, and the global simulation envelope at *α* = 5% by dotted lines. The black horizontal line at *g*_12_(*r*) = 1 is the expectation for spatial independence between congeners

Although the three *Myrcia* species occur in similar environments in the plot, we found no spatial associations indicating habitat filtering to the same favorable patches, or spatial dissociation as evidence of interspecific competition. The same size class of the species studied was, in most cases, spatially independent; that is, the observed *g*_12_(*r*) fell within the simulation envelopes of the null model of independence. The only exception was medium trees of *M. multiflora* and *M. racemosa*, which were spatially associated (Appendix S3). This association could be explained by a shared habitat association of the two congener species, as *g*_12_(*r*) fell within the simulation envelopes when we used the parametric intensity function *λ_h_*(*x*, *y*) to generate the null model patterns (Figure 3).

### 3.4 Spatial distribution of size between *Myrcia* species

**FIGURE 4:**
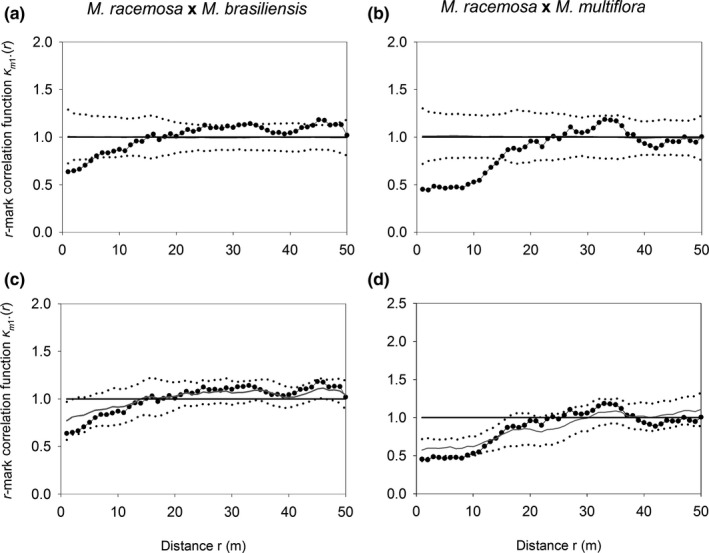
Mean stem diameter at soil height (DSH), as measured by the bivariate *r*‐mark correlation function *κ_m_*_1_.(*r*), between medium trees of *Myrcia racemosa* in relation to *Myrcia brasiliensis* and *Myrcia multiflora* (pairwise) sampled in a 1‐ha plot of white‐sand flooded forest, southeastern Brazil. The mean DSH was compared to the independent marking null model (a and b) and to the local random marking null model with *R* = 25 m (c and d). The observed *κ_m_*_1_.(*r*) is represented by closed circles, the mean *κ_m_*_1_.(*r*) of 199 simulations by gray solid lines, and the global simulation envelope at *α* = 5% by dotted lines. The horizontal line at *κ_m_*_1_.(*r*) = 1 is the expectation for independence between DSH and distance between congeners

We also found no evidence of interspecific competition in relation to the size of the three species. The sizes of trees were in most cases not influenced by the presence of nearby congeners of the same size class, as indicated by results of the independent marking null model. The only exception was medium‐sized trees of *M. racemosa*, which showed smaller DSH near individuals of *M. brasiliensis* (< 6 m) and of *M. multiflora* (< 13 m). However, this effect was caused by systematic size differences caused by unfavorable environmental conditions, as the observed *κ_m_*_1_.(*r*) fell within the simulation envelopes of the local random marking null model (Figure 4).

### 3.5 Spatial relationship and spatial distribution of size between each *Myrcia* species and heterospecifics

The distribution of the individuals in the largest size class of the three *Myrcia* species was spatially independent from the distribution of the individuals belonging to each of the 11 abundant species in the plot (*n* ≥ 30). Additionally, size of the larger *Myrcia* trees was independent from the distance of nearby abundant heterospecifics. All abundant species occur in both flooded and dry areas of the plot, and include five Myrtaceae species, from which three belong to *Myrcia* (Appendix S2).

### 3.6 Spatial relationship between *Myrcia* conspecifics of different sizes

Only *M. multiflora* showed spatial associations between different size classes, which were best explained by dispersal limitation for small trees in relation to large trees, and combination of dispersal limitation and habitat filtering for medium size trees in relation to large trees (Table 3). For *M. brasiliensis* and *M. racemosa*, small and medium trees were spatially independent of large trees, but for the small–large tree combination of *M. racemosa*, the habitat filtering model received similar support as the independence model (Table 3). The parameter *σ* that resulted in the best fit for the models of dispersal limitation was 20 m for *M. brasiliensis*, 9 m for *M. multiflora*, and 15 m for *M. racemosa*.

**TABLE 3:** Akaike's information criterion values for four competing hypothesis explaining spatial relationships at distance *r* = 1–50 m between small and large trees, and between medium and large trees of conspecifics of three *Myrcia* species sampled in a 1‐ha plot of white‐sand flooded forest, southeastern Brazil

Conspecifics interactions	Independence	Association due to dispersal limitation	Association due to habitat filtering	Association due to the combination of dispersal limitation and habitat filtering	
Intensity function	*λ*(*x,y*)	*λ*_*d*_(*x,y*)	*λ*_*h*_(*x,y*)	$\sqrt{\lambda_{d}\cdot \lambda_{h}}$	Fitted value of *σ*
*M. brasiliensis*					
Large × Small trees	**−158.58**	−152.78	−144.56	−146.9	20 m
Large × Medium trees	**−119.88**	−85.48	−107.06	−89.7	20 m
*M. multiflora*					
Large × Small trees	−121.28	**−179.66**	−144.78	−158.6	9 m
Large × Medium trees	−106.12	−150.42	−159.78	**−162.16**	9 m
*M. racemosa*					
Large × Small trees	**−159.58**	95.52	−159.74	−142.3	15 m
Large × Medium trees	**−171.90**	−130.62	−163.84	−159.6	15 m

Note

Bold indicates the best fitted model. Bold face indicates the most parsimonious model(s).

## 4 DISCUSSION

In this study, habitat filtering was the most important process driving the local distribution of three *Myrcia* species in a white‐sand flooded tropical forest, as species distribution showed associations, albeit of different strength, to environmental variables. We did not detect spatial patterns that are consistent with interspecific competition between the three species studied and between each *Myrcia* species and other co‐occurring species in the plot, that is, spatial segregation and smaller size of nearby neighbors. Additionally, the three *Myrcia* were spatially independent, but responded to differences in the environment. Last, dispersal limitation only led to spatial associations of different size classes for one species studied. Like our results, many studies on topo‐edaphic variation in tropical forests (e.g., Baldeck et al., 2012), including flooded areas (Baraloto et al., 2007; Oliveira et al., 2014), have shown that habitat filtering is an important ecological process driving community structure.

### 4.1 Relationship between the distribution of the three *Myrcia* species and environmental variables

The distribution of the three congeneric species showed somewhat similar associations to environmental variables, which are expected due to their close phylogenetic relationship (Losos, 2008). In general, the distribution of the three *Myrcia* species was related to lower elevation and terrain slope, and higher canopy openness. Flooding is expected to be more frequent, last for a longer time, and/or reach higher depths at lower parts of the study plot due to periodic water table flood (Diniz, 2009). As shown in Figure 2, the lower part of the study plot does present higher soil moisture and flooding depth. Therefore, we believe elevation is a good surrogate for flooding at the study site. Additionally, we detected relationships between the distribution of small and large trees with higher soil moisture for *M. multiflora*, which reinforces our hypothesis that the three congeneric species occur predominantly in patches subject to flooding within the plot.

We probably found no further relationships between species distribution and flooding depth and/or soil moisture because both variables were measured only once and may thus not be able to capture flooding seasonality properly. The distribution of the three congeneric species was also restricted to better‐lit patches, where individuals are likely to show higher survival probability, growth rate, and fecundity (Dahlgren & Ehrlén, 2009). Interestingly, we were able to detect the influence of temporal changes in the light environment on the distribution of different size classes of the three *Myrcia* species. Although smaller trees were correlated with recent and/or past light environment (canopy openness in 2017 and 2008, respectively), large trees were correlated only with the past light environment, which probably reflects more closely the conditions of the time when plants were smaller.

*Myrcia brasiliensis*, which is well distributed in the study plot, showed weak associations to the environmental variables. Conversely, the other two species, whose distributions follow a flood–dry gradient, showed stronger associations to elevation. For the three species, environmental requirements shifted across demographic stages of plant development, as also observed for other species (e.g., Comita et al., 2007). For *M. brasiliensis*, the light environment was important for small trees (Poorter, 2007), while large trees no longer depended on light, as the species occupies the first canopy stratum and also occurs as an emergent tree (Valladares et al., 2016). As individuals grow in better‐lit environments, some become restricted to patches subject to flooding. On the other hand, *M. racemosa* showed an opposite pattern, whereby smaller trees get restricted to patches subject to flooding and later on development the past light environment becomes detectable on the distribution of large trees. The shift in environmental requirements likely explains the change in distribution from small and medium trees (flood–dry gradient) to large trees (from northwest to southeast of the plot) for *M. racemosa*. *Myrcia multiflora* showed an intermediate pattern, with species distribution restricted to patches subject to flooding from early to late development, and light influencing the distribution of small and medium trees.

Even though flooding is the most important environmental characteristic driving species distribution in the plot, the fundamental niche of the three *Myrcia* species potentially encompasses a much wider variation of conditions than the environments of the study site, as the species occur in different vegetation types and are not restricted to areas subject to flooding at the geographic scale (Appendix S1). Thus, in the white‐sand forest studied, the species can use the most common environmental condition (niche position, Marino et al., 2019) and patches subject to flooding may work as areas of refuge from strong competitors and/or natural enemies (Baraloto et al., 2007), which drives the species’ realized niche. The same reasoning may apply to other species in the community, because most occur in both flooded and dry patches within the study plot (Appendix S2). We suggest future studies should evaluate flood tolerance of the different species of the community and the presence of natural enemies to better understand the spatial distribution constraint in a gradient of soil moisture in flooded forests.

### 4.2 Spatial relationship and spatial distribution of size between *Myrcia* species and between each *Myrcia* species and heterospecifics

The overall association of the three *Myrcia* species to patches subject to flooding let us expect spatial association between congeners. Nevertheless, only medium trees of *M. multiflora* and *M. racemosa* were spatially associated due to the same habitat association. All other congener pairs showed independence, as well as pairs between the three focal *Myrcia* species and the abundant species in the plot. Interestingly, the abundant species include other *Myrcia* and Myrtaceae species, and so our results suggest that plant–plant interactions, even among closely related species, are not the main drivers structuring of our white‐sand tropical forest community. It is possible that the three focal *Myrcia* species preferentially use areas subject to flooding in the plot as a refuge from natural enemies such as pathogens, which are important mortality agents of tropical plant species (Comita et al., 2014; Terborgh, 2012).

Spatial independence between species has been frequently shown for tropical tree species (Wang et al., 2016; Wiegand et al., 2012). One explanation is that positive and negative interactions between species cancel each other's effects out, resulting in spatial patterns that appear overall to be independent (Punchi‐Manage et al., 2015). Also, according to the stochastic dilution hypothesis, independence should be more common in more species‐rich forests due to the large variability in the identity of neighbored trees that inhibit the detection of the spatial arrangement of plants directed by interactions between species (Wang et al., 2016).

The apparent spatial independence between congeners (and between *Myrcia* and other heterospecifics) is in line with the independence between tree size and distance between species when we considered environmental heterogeneity in our analysis (i.e., local random marking null model). The evaluation of plant performance in greenhouse experiments and functional traits related to resource acquisition could help to elucidate whether our three focal *Myrcia* species use resources differently, as expected by niche differentiation of sympatric species with similar environmental requirements due to strong competition in the past (Connell, 1980).

### 4.3 Spatial relationship between *Myrcia* conspecifics of different sizes

Even though dispersal limitation is common in tropical forests (Hubbell, 2005) and habitat filtering is expected to influence species distribution in naturally disturbed environments (Baraloto et al., 2007), we found that the combination of these two processes only best explained the observed spatial associations between small/medium and large trees of *M. multiflora*. Dispersal limitation drives the initial spatial relationship between small and large *M. multiflora* trees, and as small trees grow into medium‐sized trees, habitat filtering gains an importance for this species, which was also indicated by the stronger associations (i.e., higher *Z* values) of medium and large trees to environmental variables. Note that we estimated for *M. multiflora* the smallest width of the kernel function (*σ* = 9 m vs. 15 m and 20 m for the other two congeners) of the dispersal limitation model. Although it is consistent with species height (occupation in different canopy strata), we cannot exclude the possibility that the size of our plot is too small to allow for the detection of the effects of dispersal limitation for *M. racemosa* and especially *M. brasiliensis*, which shows the largest tree height, fruit diameter and width of the kernel function (*σ* = 20 m) among the three species studied. Because larger fruits can be dispersed by larger animals (Janson, 1983; Wheelwright, 1985), which have larger home ranges (Kelt & Van Vuren, 2001), seeds of *M. brasiliensis* are likely to be dispersed further from parent plants relative to the other two congeners and thus show lower dispersal limitation (Seidler & Plotkin, 2006). Therefore, a larger study site would be needed to detect spatial patterns consistent with dispersal limitation at larger spatial scales.

Spatial independence between small and large trees was also found by Getzin et al. (2014) on Barro Colorado Island, Panama. The authors suggested that this pattern could result from habitat association masked by unpredictable dispersal events created by disperser movement behavior or from the uncoupling of the positive association of trees expected from dispersal limitation due to high mortality of seeds and early developmental stages caused by conspecific negative‐density dependence (CNDD). Because CNDD is widespread in tropical forests (LaManna et al., 2017), a future project is to address whether CNDD of the three *Myrcia* species uncouples the spatial distribution patterns of trees from the seed deposition patterns. Additionally, some studies have shown that phylogenetically related neighbors increase mortality of a focal species (Bagchi et al., 2010; Metz et al., 2010; Paine et al., 2012; Zhu et al., 2015), so our three *Myrcia* species could show phylogenetic density dependence. Last, we still lack knowledge on how flooding may affect CNDD, which is especially important in the face of future environmental changes.

## 5 CONCLUSIONS

In this study, we employed several spatial point process models to simultaneously investigate important ecological processes that are hypothesized to maintain species coexistence in tropical forests, that is, habitat filtering, interspecific competition, and dispersal limitation. Using data from congeneric species as a model in a naturally disturbed environment, we demonstrated that habitat filtering to areas subject to flooding is the most important ecological process driving the local distribution of these species in a white‐sand flooded tropical forest. In this type of vegetation, there seems to be a clear division of tree species into either flood‐tolerant or flood‐intolerant (Oliveira et al., 2014). However, climate change and other anthropic pressures can affect species coexistence by providing new habitat conditions and so understanding how habitat filtering acts in forests subject to flooding potentially aids mitigation plans of environmental changes (Kraft et al., 2015). Given that *restinga* is highly threatened in Brazil (Alho et al., 2002), the preservation of its different habitats is of utmost importance to the maintenance of high species richness and functional strategies.

## CONFLICT OF INTEREST

None declared.

## AUTHOR CONTRIBUTIONS

**Kelly F. O. Ribeiro:** Conceptualization (equal); formal analysis (lead); funding acquisition (lead); methodology (equal); supervision (lead); writing – original draft (equal); writing – review and editing (equal). **Valéria F. Martins:** Conceptualization (equal); formal analysis (supporting); funding acquisition (supporting); methodology (equal); supervision (supporting); writing – original draft (equal); writing – review and editing (equal). **Thorsten Wiegand:** Conceptualization (equal); formal analysis (lead); funding acquisition (supporting); methodology (equal); software (lead); supervision (supporting); writing – original draft (supporting); writing – review and editing (supporting). **Flavio A. M. Santos:** Conceptualization (equal); formal analysis (supporting); funding acquisition (lead); methodology (equal); supervision (lead); writing – original draft (supporting); writing – review and editing (supporting).

## Supporting information

Appendix S1‐S3

## Data Availability

The data are available from Knowledge Network for Biocomplexity (KNB) repository at https://doi.org/10.5063/X928QD.
