# Supplementary material for: Habitat filtering drives the local distribution of congeneric species in a Brazilian white‐sand flooded tropical forest
Source: Ecol Evol. 2021 Jan 17;11(4):1797–813. doi: 10.1002/ece3.7169 (PMC7882942; doi:10.1002/ece3.7169)

Appendix S1. Geographical distribution of *Myrcia brasiliensis*, *M. multiflora* and *M. racemosa* in Brazil. Data obtained from the speciesLink platform (2019). The green crosses are the coordinates reported by biological collections; the delimited regions represent the different phytogeographical domains in Brazil.

Appendix S2. Tree and palm species, their abundance and occurrence in dry, flooded or both environments in a 1-ha plot of white-sand flooded forest, southeastern Brazil. Bold indicates abundant species (> 30 individuals). Environment indicates that all individuals of each species were located in exclusively flooded, dry or both subplots (10 x 10 m).

| **Family** | **Species** | **Abundance** | **Environment** |
| --- | --- | --- | --- |
| Annonaceae | *Guatteria australis* | 3 | both |
| Annonaceae | ***Guatteria*** **sp. 4** | **41** | both |
| Aquifoliaceae | *Ilex integerrima* | 7 | both |
| Araliaceae | ***Schefflera angustissima*** | **31** | both |
| Arecaceae | ***Euterpe edulis*** | **142** | both |
| Bignoniaceae | ***Jacaranda puberula*** | **78** | both |
| Celastraceae | ***Maytenus littoralis*** | **51** | both |
| Celastraceae | *Maytenus* sp. | 9 | both |
| Chrysobalanaceae | *Hirtella hebeclada* | 2 | both |
| Clusiaceae | ***Garcinia gardneriana*** | **36** | both |
| Clusiaceae | *Kielmeyera petiolaris* | 26 | both |
| Elaeocarpaceae | *Sloanea guianensis* | 7 | both |
| Erythroxylaceae | *Erythroxylum* sp. | 6 | both |
| Euphorbiaceae | ***Alchornea triplinervia*** | **67** | both |
| Fabaceae | *Andira fraxinifolia* | 23 | both |
| Fabaceae | *Inga edulis* | 3 | both |
| Fabaceae | *Inga subnuda* | 20 | both |
| Lacistemataceae | *Lacistema lucidum* | 5 | both |
| Lacistemataceae | *Lacistema pubescens* | 4 | both |
| Lauraceae | *Aniba viridis* | 2 | both |
| Lauraceae | *Nectandra oppositifolia* | 26 | both |
| Malpighiaceae | *Byrsonima ligustrifolia* | 2 | both |
| Melastomataceae | *Miconia prasina* | 1 | both |
| Meliaceae | ***Guarea macrophylla subsp. tuberculata*** | **71** | both |
| Myristicaceae | *Virola bicuhyba* | 3 | both |
| Myrtaceae | *Calyptranthes lucida* | 4 | both |
| Myrtaceae | *Eugenia astringens* | 9 | both |
| Myrtaceae | *Eugenia brasiliensis* | 12 | both |
| Myrtaceae | *Eugenia fusca* | 5 | both |
| Myrtaceae | ***Eugenia verticillata*** | **41** | both |
| Myrtaceae | *Marlierea obscura* | 5 | both |
| Myrtaceae | ***Marlierea tomentosa*** | **33** | both |
| Myrtaceae | ***Myrcia brasiliensis*** | **77** | both |
| Myrtaceae | ***Myrcia multiflora*** | **49** | both |
| Myrtaceae | ***Myrcia racemosa*** | **152** | both |
| Nyctaginaceae | *Guapira opposita* | 25 | both |
| Peraceae | ***Pera glabrata*** | **248** | both |
| Phyllanthaceae | *Hieronyma alchorneoides* | 5 | both |
| Primulaceae | *Myrsine venosa* | 24 | both |
| Proteaceae | *Euplassa cantareirae* | 19 | both |
| Proteaceae | *Roupala montana var. brasiliensis* | 3 | both |
| Rubiaceae | *Faramea pachyantha* | 15 | both |
| Rubiaceae | *Genipa infundibuliformis* | 5 | both |
| Sapindaceae | *Cupania oblongifolia* | 3 | both |
| Sapindaceae | *Matayba elaeagnoides* | 3 | both |
| Urticaceae | *Coussapoa microcarpa* | 5 | both |
| Anacardiaceae | *Tapirira guianensis* | 9 | flooded |
| Apocynaceae | *Aspidosperma* sp. | 3 | flooded |
| Apocynaceae | *Tabernaemontana laeta* | 1 | flooded |
| Aquifoliaceae | *Ilex theezans* | 6 | flooded |
| Araliaceae | *Oreopanax capitatus* | 1 | flooded |
| Arecaceae | *Astrocaryum aculeatissimum* | 4 | flooded |
| Chrysobalanaceae | *Couepia venosa* | 2 | flooded |
| Clusiaceae | *Calophyllum brasiliense* | 9 | flooded |
| Clusiaceae | *Clusia criuva subsp. parviflora* | 1 | flooded |
| Fabaceae | *Abarema brachystachya* | 3 | flooded |
| Fabaceae | *Balizia pedicellaris* | 1 | flooded |
| Fabaceae | *Fabaceae* sp. 4 | 1 | flooded |
| Fabaceae | *Inga* sp. | 1 | flooded |
| **Family** | **Species** | **Abundance** | **Environment** |
| Fabaceae | *Lonchocarpus cultratus* | 2 | flooded |
| Fabaceae | *Ormosia arborea* | 4 | flooded |
| Fabaceae | *Swartzia simplex var. grandiflora* | 3 | flooded |
| Fabaceae | *Tachigali denudata* | 1 | flooded |
| Lauraceae | *Ocotea rariflora* | 1 | flooded |
| Melastomataceae | *Miconia dodecandra* | 1 | flooded |
| Melastomataceae | *Miconia rigidiuscula* | 3 | flooded |
| Melastomataceae | *Miconia* sp. | 1 | flooded |
| Melastomataceae | *Miconia* sp. 1 | 1 | flooded |
| Melastomataceae | *Miconia* sp. 3 | 1 | flooded |
| Moraceae | *Sorocea jureiana* | 2 | flooded |
| Myrtaceae | *Calyptranthes concinna* | 12 | flooded |
| Myrtaceae | *Eugenia badia* | 1 | flooded |
| Myrtaceae | *Eugenia copacabanensis* | 1 | flooded |
| Myrtaceae | *Eugenia handroana* | 3 | flooded |
| Myrtaceae | *Eugenia monosperma* | 1 | flooded |
| Myrtaceae | *Eugenia mosenii* | 1 | flooded |
| Myrtaceae | *Eugenia speciosa* | 3 | flooded |
| Myrtaceae | *Marlierea racemosa* | 1 | flooded |
| Myrtaceae | *Marlierea spectabilis* | 1 | flooded |
| Myrtaceae | *Myrcia spectabilis* | 4 | flooded |
| Myrtaceae | *Myrtaceae* sp. 10 | 1 | flooded |
| Myrtaceae | *Syzygium jambos* | 6 | flooded |
| Primulaceae | *Myrsine coriacea* | 5 | flooded |
| Rosaceae | *Prunus myrtifolia* | 1 | flooded |
| Rubiaceae | *Amaioua intermedia* | 13 | flooded |
| Rubiaceae | *Posoqueria latifolia* | 7 | flooded |
| Sapindaceae | *Cupania vernalis* | 1 | flooded |
| Sapotaceae | *Chrysophyllum flexuosum* | 1 | flooded |
| Theaceae | *Laplacea fruticosa* | 2 | flooded |
| Urticaceae | *Cecropia glaziovii* | 1 | flooded |
| Annonaceae | *Guatteria* sp. | 2 | dry |
| Aquifoliaceae | *Ilex dumosa* | 1 | dry |
| Elaeocarpaceae | *Sloanea hirsuta* | 1 | dry |
| Euphorbiaceae | *Margaritaria nobilis* | 2 | dry |
| Lamiaceae | *Vitex cymosa* | 1 | dry |
| Lauraceae | *Endlicheria paniculata* | 1 | dry |
| Lauraceae | *Persea willdenovii* | 1 | dry |
| Melastomataceae | *Miconia latecrenata* | 22 | dry |
| Monimiaceae | *Mollinedia schottiana* | 1 | dry |
| Moraceae | *Ficus insipida* | 1 | dry |
| Moraceae | *Sorocea hilarii* | 1 | dry |
| Myrtaceae | *Campomanesia guaviroba* | 1 | dry |
| Myrtaceae | *Eugenia multicostata* | 1 | dry |
| Myrtaceae | *Marlierea sylvatica* | 1 | dry |
| Myrtaceae | *Myrcia pubipetala* | 3 | dry |
| Myrtaceae | *Myrcia splendens* | 3 | dry |
| Myrtaceae | *Psidium cattleianum* | 1 | dry |
| Olacaceae | *Heisteria silvianii* | 1 | dry |
| Polygonaceae | *Coccoloba glaziovii* | 4 | dry |
| Rubiaceae | *Coussarea meridionalis var. porophylla* | 1 | dry |

Appendix S3. Spatial relationship, as measured by the bivariate pair correlation function *g*_12_(*r*), between individuals in the same size class of three *Myrcia* species (pairwise) sampled in a 1-ha plot of white-sand flooded forest, southeastern Brazil. The observed *g*_12_(*r*) is represented by closed circles, the mean *g*_12_(*r*) of 199 pattern reconstruction simulations of the point pattern underlying the independence null model by grey solid lines, and the global simulation envelope at α = 5% by dotted lines. The location of the first species was randomized while the location of the second species was kept fixed. The black horizontal line at *g*_12_(*r*) = 1 is the expectation for spatial independence between congeners without large-scale habitat association.


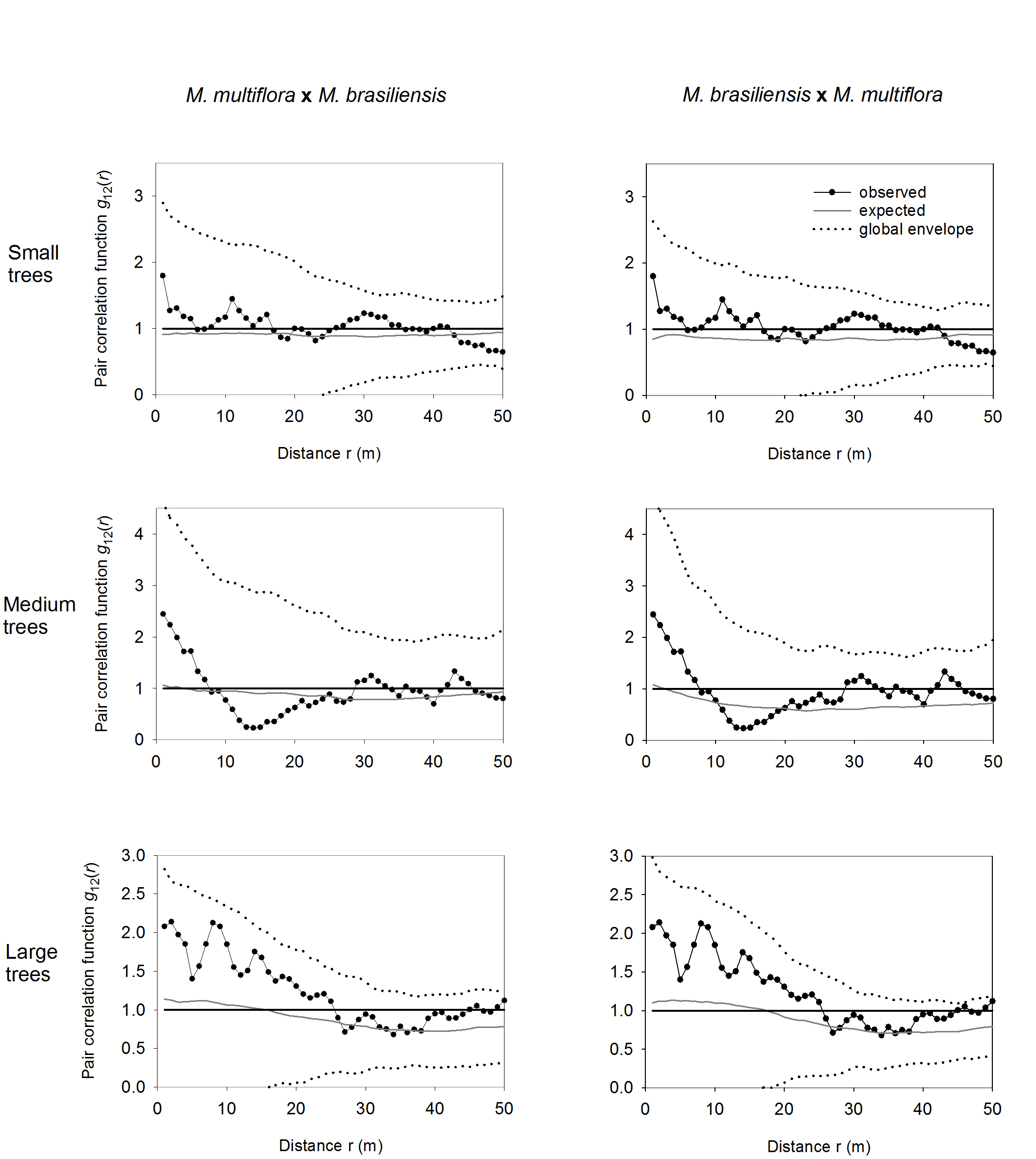

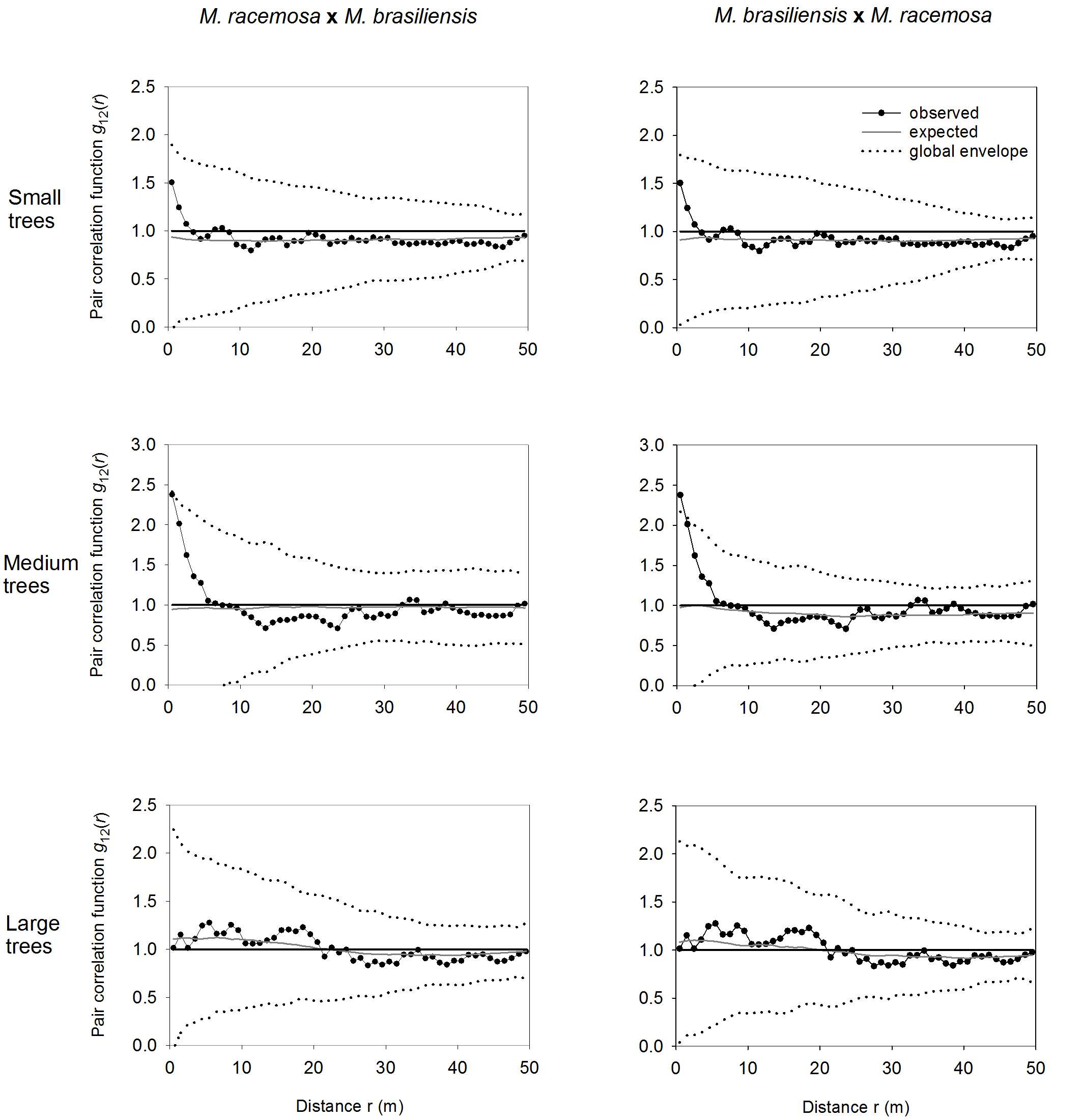

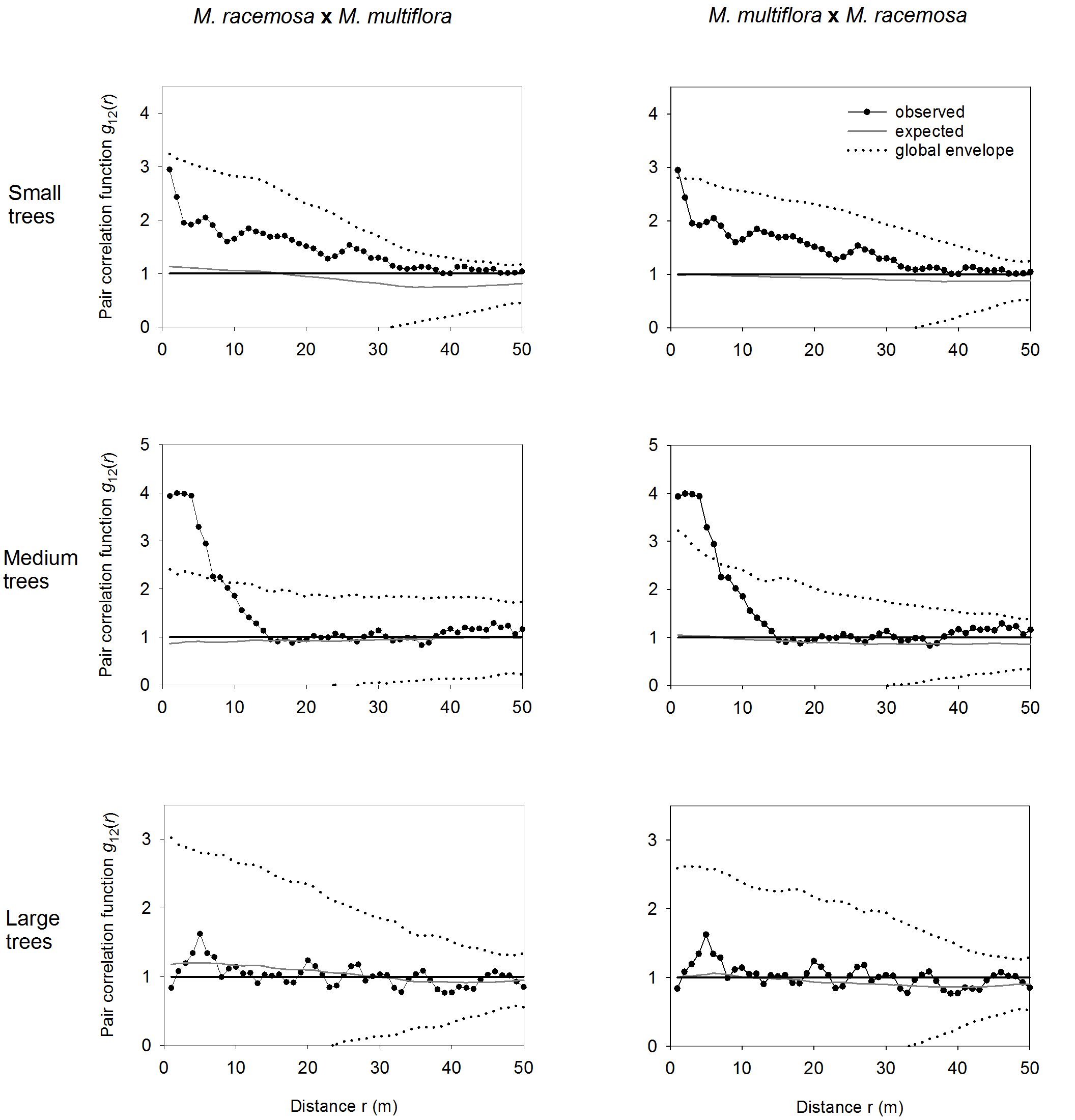

Supplement: Supplementary file 1 — Appendix S1‐S3 [file ECE3-11-1797-s001.docx]
